# Silver Nanoparticles and Their Antibacterial Applications

**DOI:** 10.3390/ijms22137202

**Published:** 2021-07-04

**Authors:** Tamara Bruna, Francisca Maldonado-Bravo, Paul Jara, Nelson Caro

**Affiliations:** 1 Centro de Investigación Austral Biotech, Facultad de Ciencias, Universidad Santo Tomás, Avenida Ejército 146, Santiago 8320000, Chile; francisca.maldonado@ug.uchile.cl; 2 Departamento de Química, Facultad de Ciencias, Universidad de Chile, Las Palmeras 3425, Ñuñoa, Santiago 7800003, Chile; pjara@uchile.cl

**Keywords:** silver nanoparticles, antibacterial activity, cytotoxicity, medical applications, antibiotic alternative

## Abstract

Silver nanoparticles (AgNPs) have been imposed as an excellent antimicrobial agent being able to combat bacteria in vitro and in vivo causing infections. The antibacterial capacity of AgNPs covers Gram-negative and Gram-positive bacteria, including multidrug resistant strains. AgNPs exhibit multiple and simultaneous mechanisms of action and in combination with antibacterial agents as organic compounds or antibiotics it has shown synergistic effect against pathogens bacteria such as *Escherichia coli* and *Staphylococcus aureus*. The characteristics of silver nanoparticles make them suitable for their application in medical and healthcare products where they may treat infections or prevent them efficiently. With the urgent need for new efficient antibacterial agents, this review aims to establish factors affecting antibacterial and cytotoxic effects of silver nanoparticles, as well as to expose the advantages of using AgNPs as new antibacterial agents in combination with antibiotic, which will reduce the dosage needed and prevent secondary effects associated to both.

## 1. Introduction

Silver in all its forms has been historically used as an antimicrobial agent by itself or combined with other technologies [1]. This metal has been studied to take advantage of its ability to inhibit bacterial growth by incorporating it as silver nitrate or silver sulfadiazine in creams and dressings to treat burns and ulcers, in food packaging to prevent contamination, in home appliances as refrigerators and washing machines, and several applications in the industrial area [2,3,4,5,6]. Because of the knowledge and evidence existing of the antibacterial activity of silver [7], with the emergence of nanotechnology, the exploration of the antibacterial capacity of AgNPs was an evident path.

AgNPs are defined as a nanomaterial with all its dimensions in the range of 1–100 nm. These have shown greater capacity and higher surface (area-to-volume ratio) compared to silver in its bulk form. At the nanoscale, this material exhibits unique electrical, optical, and catalytic properties, which has led to the investigation and fabrication of products for targeted drug delivery, diagnosis, detection, and imaging [1,8]. However, it is the exceptional antibacterial activity exhibited by AgNPs that has focused the attention of researchers and industries on this nanomaterial. AgNPs have shown antimicrobial activity against a variety of infectious and pathogenic microorganisms, including multidrug-resistant bacteria [9,10].

The enhanced antibacterial activity of Ag at the nanoscale has been most valuable in medical and healthcare areas, where the incorporation of AgNPs into hundreds of products has been studied, including surgical and food handling tools, clothing, cosmetics, dental products, catheters, and dressings [11,12,13,14]. The potential of AgNPs as antibiotics is related to their various mechanisms of action, which attack microorganisms in multiple structures at a time and give them the ability to kill various types of bacteria [15].

Currently, investigations related to the development of new antibiotics are difficult processes; they require years of studying the efficacy and safety of the agents, consuming high amounts of time and resources, while infections caused by multi-resistant microorganisms keep growing and causing deaths worldwide [16]. AgNPs, along with other nanomaterials, have been studied in the defined post-antibiotic era to search for new agents that can help combat pathogenic microorganisms without promoting the appearance of new resistances [16]. As infections caused by antibiotic-resistant microorganisms are a matter of global concern, AgNPs arise as an excellent alternative as they can be applied to prevent infections caused by these microorganisms, decontaminate medical supplies, and even combat infections in course [16,17]. As an antibiotic alternative, this application has been broadly studied in recent years with the objective of developing new bactericidal products for decontamination or infection treatments taking advantage of the already established knowledge about their efficiency even against multidrug resistant organisms [18].

The attention captured by AgNPs is reflected in the figures of high demand and investment in research related to them. In the last 15 years, the market of AgNPs has been growing steadily, with an estimated production of more than 500 tons of nanoparticles per year to supply the different industries’ demands [19]. Due to the growth of the nanoparticle market worldwide and the current offer of products with incorporated nanoparticles, the study of their biological activity and safety has become a matter of issue, along with the elucidation of their exact mechanisms of action in bacterial and mammalian cells [20].

One of the factors of major consideration is the toxicity that nanoparticles could have for human and environmental health, given that their size, which can be considered their main advantage, is also what attributes to them the possibility of crossing the defense barriers in organisms, being able to induce mild to chronic toxic effects after their accumulation. The biological effects and factors that influence AgNPs activity will be discussed in this review. Research addressing the antibacterial and cytotoxic effects of AgNPs will be presented to provide background information regarding the interactions of AgNPs with biological systems and the parameters affecting these. The antibacterial mechanisms and investigations related to pathogenic bacteria are presented with the objective of highlighting the advantages of using AgNPs-based products to combat infections caused by these bacteria. For this last purpose, some examples of current biomedical applications of AgNPs are presented.

## 2. AgNPs Synthesis

Different approaches classified as bottom-up or top-down methods are used to synthesize metallic nanoparticles [1]. Top-down approaches synthesize nanoparticles from metallic silver in solid or aerosolized state down into the nanoscale, obtaining stable AgNPs. In this category are the physical methods such as ball milling, laser ablation, and sputtering [11,21,22,23]. On the other hand, bottom-up approaches consist of the nanostructuring and stabilization of silver atoms through different methods in order to form nanoparticles. Bottom-up methods include chemical and biological techniques applied to synthesize nanoparticles [1,12].

Physical methods are commonly used to obtain large quantities of nanoparticles, and depending on the technique, it may provide highly pure nanoparticles. However, these methods usually require large amounts of energy, expensive instrumentation, and high pressure and temperature conditions [19,24].

Chemical methods for the synthesis of AgNPs include electrochemical, sol-gel, and chemical reduction. They allow obtaining nanoparticles with a defined spherical shape and are considered low cost [19,25,26]. These methods require a metallic precursor, a reducing agent, and a stabilizing agent, thus they are considered simple to perform and scalable. However, by requiring the use of these agents, toxic reagents or solvents are often incorporated, which can produce polluting or hazardous waste [12,27].

The third method of biological synthesis of nanoparticles includes all forms of synthesis involving components of biological origin or organisms themselves, either fungal or bacterial mediated synthesis or synthesis using natural extracts as reagents [28,29,30]. Biologically prepared AgNPs have demonstrated high solubility, yield, and stability [31]. However, the use of organisms or reagents of biological origin adds complexity to this synthesis process, and the fact that compounds with a high capacity to stabilize and act as a reducing agent in the process must be used. Nevertheless, this is considered one of the most promising methods in view that it is low cost and there is a wide variety of natural resources to be used that contribute to reducing the potential toxicity of nanoparticles [24].

**Figure 1:**
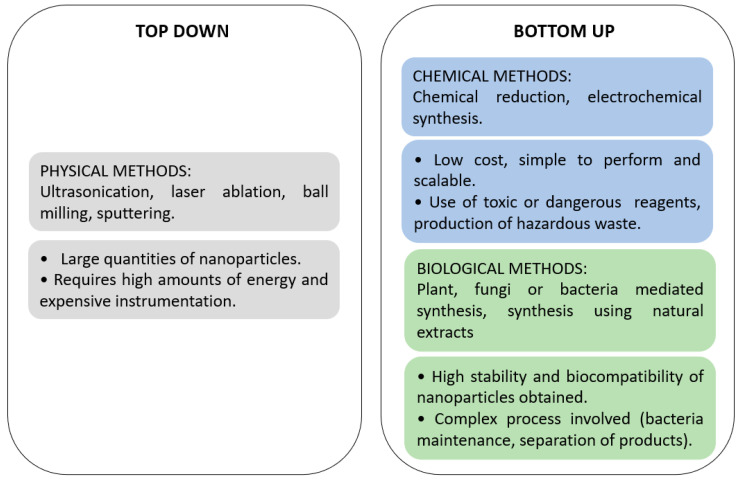
Synthesis methods for AgNPs preparation and its characteristics.

Each approach requires different techniques, instrumentation, and conditions that determine their difficulties and advantages over the others. A resume of the synthesis methods is presented in Figure 1. The synthesis method used will affect properties such as size, stability, and biological effects of the nanoparticles defining their chemical surface and ion release capacity. Therefore, the synthesis will be determinant in their biological activity and possible toxicity [27,32].

Considering this, the method chose to synthesize AgNPs with potential use in antibacterial products or therapies should be selected in order to optimize the antibacterial activity of the nanoparticles while decreasing the cytotoxic effects. The effect of different factors that can be controlled during the synthesis of nanoparticles on the antibacterial and cytotoxic potential of AgNPs are reviewed in the next sections.

## 3. Antibacterial Action of AgNPs

AgNPs have exhibited highly antibacterial action against multiple Gram-positive and Gram-negative bacteria [33]. However, the exact mechanism by which they exert inhibitory growth or bactericidal activity has not been fully elucidated yet. The existing experimental evidence supports different mechanisms that consider the physicochemical properties of AgNPs, such as size and surface, which allow them to interact or even pass through cell walls or membranes and directly affect intracellular components.

### 3.1. Mechanisms of Antibacterial Action

Currently, the literature supports principally three mechanisms that have been observed together or separately, by which AgNPs exert their antibacterial action [10,34,35]. The first one postulates that AgNPs act at a membrane level as they are able to penetrate the outer membrane, accumulating in the inner membrane where the adhesion of the nanoparticles to the cell generates their destabilization and damage, increasing membrane permeability and inducing leakage of cellular content and subsequently its death [36,37]. It is also evidenced that AgNPs can interact with sulfur-containing proteins in the cell wall of bacteria, an interaction that may cause structural damage leading to cell wall rupture.

The second mechanism proposes that nanoparticles not only can break and cross the cell membrane, altering its structure and permeability but can also enter the cell where it has been suggested that, due to its properties, AgNPs will have an affinity to interact with sulfur or phosphorus groups, present in intracellular content such as DNA and proteins altering their structure and functions. In the same manner, they may alter the respiratory chain in the inner membrane by interacting with thiol groups in the enzymes inducing reactive oxygen species and free radicals, generating damage to intracellular machinery and activating the apoptosis pathway. A third mechanism that is proposed to occur in parallel with the two others is the release of silver ions from the nanoparticles, which due to their size and charge, can interact with cellular components altering metabolic pathways, membranes, and even genetic material [37,38,39,40,41,42].

### 3.2. Factors Affecting Antibacterial Activity of AgNPs

Along with the elucidation of the mechanistic aspects of AgNPs antibacterial activity, it has also been established how the properties of these nanoparticles, namely chemical size, charge, and surface, influence their antibacterial capacity.

Related to the size, Lu et al. [43] addressed its effect in the antibacterial activity of AgNPs against the bacteria responsible for caries and periodontal diseases. AgNPs of 5, 15, and 55 nm were synthesized by chemical reduction with polyvinylpyrrolidone (PVP) and their antibacterial activity against *E. coli, Fusobacterium nucleatum, Streptococcus mutans, Streptococcus sanguis, Streptococcus mitis*, and *Aggregatibacter actinomycetemcomitans* was evaluated. Revealing that the 5 nm nanoparticles possessed a better antibacterial effect, with minimum inhibitory concentrations (MIC) between 25 and 50 μg/mL for the microorganisms tested, with the exception of *E. coli* strain assayed, which MIC value was 6 μg/mL. This big difference in relation to MICs of the other microorganisms tested was attributed to the aerobic character of *E. coli*, versus the other pathogenic bacteria that were anaerobic. It is hypothesized in the research that this effect may be due to oxidation of AgNPs in aqueous media when exposed to air, the reaction that reduces its antibacterial capacity [43].

In another investigation, AgNPs of different sizes were synthesized using the same agents and general protocol but changing the conditions of pH and proportions of reducing and stabilizing agents in the reactions. Then the bactericidal and bacteriostatic capacity of nanoparticles between 5 to 100 nm were evaluated against Gram-negative and Gram-positive bacteria [44]. The MIC obtained varied between 20 to 110, 60 to 160, 30 to 120, and 70 to 200 μg/mL for two *E. coli* strains, *Bacillus subtilis*, and *S. aureus*, being the first value corresponding to the smaller nanoparticles (5 nm) and the second corresponding to the bigger nanoparticles (100 nm). In addition, bactericidal concentrations were found to be from 30 to 140 μg/mL for all the strains studied, but *S. aureus* where minimal bactericidal concentration (MBC) was higher than 200 μg/mL. As shown in the results of MIC, the antibacterial activity was highly dependent on size, relation attributed to the larger surface area of the smaller nanoparticles, available to direct contact with the bacterial cell.

A third study can be highlighted in relation to the size effect, in which 5 different AgNPs were prepared by chemical reduction, and their inhibitory activity against *E. coli* and *Pseudomonas aeruginosa* was evaluated. The analysis revealed that the smallest nanoparticles (15 to 50 nm) gave rise to an inhibition halo of 8 mm growth for *P. aeruginosa* and 1.5 mm for *E. coli*, while the largest diameter nanoparticles (30 to 200 nm agglomerates) presented the lowest activity with inhibition halos of 0.8 mm for *P. aeruginosa* and 0.7 mm for *E. coli*. In a more recent investigation, the antibacterial activity of laser-generated AgNPs of various sizes was evaluated against *E. coli*. Here, it was also found an inverse correlation between antibacterial activity and the size of the AgNPs; nanoparticles of 19 nm average size showed the most effective antibacterial activity. In this case, the researchers showed that smaller AgNPs would induce more reactive oxygen species and thereby will be more effective against *E. coli* [32,45].

Addressing the influence of the charge, it has been demonstrated that positively charged NPs had greater antibacterial activity [46,47]. Research by Abbaszadegan et al. [48] suggests that the electrostatic attraction between positively charged AgNPs and negatively charged bacterial cells is necessary for the antibacterial effectiveness of the AgNPs, and this attraction is managed by the charge of the AgNPs and the microorganisms.

It is noteworthy that both size and surface characteristics are related to the release rate of silver ions from the nanoparticles. The size of the nanoparticle influences the contact area and interaction of the nanoparticle with the medium, while the charge and surface composition determine the stability of the nanoparticles [49]. Thus, it has been observed that smaller nanoparticles have a higher dissolution rate in different media, releasing silver ions in the process, which could represent an important contribution to the antibacterial effect of nanoparticles [45,50,51].

In relation to the size and charge of the nanoparticles, the stability of the products formed is also an important factor affecting the final antibacterial activity [52]. If the synthesized AgNPs have low stability, they will tend to aggregate and form bigger particles, and as has been shown, nanoparticles with bigger sizes have lower antibacterial activity. The principal actors affecting the stability of AgNPs are the charge and the coating. Principally, considering the zeta potential of nanoparticles, it has been established that AgNPs can be classified as stable when their superficial charge is superior to +30 mV or smaller than −30 mV [53] since this will prevent their agglomeration given the repulsive interactions between nanoparticles. This parameter may be defined by the synthesis process as well as the coating agent used [52].

The coating corresponds to the outermost layer of the nanoparticle, constituting the first line of interaction between the nanoparticles and the components of the medium, and it also interferes in the antibacterial activity [54]. The nanoparticle coating can be modified by adding different agents during the synthesis process or later [11,55]. Thus, according to the desired effect, nanoparticles can be synthesized with chemical agents that possess antibacterial activity by themselves, with the aim of promoting this same activity in the final nanoparticle. As it will be reviewed later, it has been studied that coating nanoparticles with polymers or organic compounds generate nanoparticles with low or no cytotoxicity against mammalian cells without reducing the toxic effects against bacteria assayed. [56,57]. For example, using chitosan for coating AgNPs, has shown high inhibitory activity against *S. aureus*, *P. aeruginosa*, and *Salmonella typhimurium*, reducing the number of colonies up to 95% after 4 h of contact [58].

The influence of the properties of AgNPs on their antibacterial activity has been experimentally demonstrated. The effect of these characteristics on the antibacterial capacity is related to the mechanisms by which AgNPs act on bacteria. It is observed that those parameters that facilitate nanoparticle-bacterial cell interactions, as well as ingress, will be those that enhance antimicrobial activity. The fact that the nanoparticles exhibit variable antibacterial activity according to their properties allows their manipulation and fabrication based on the final desired objectives of the nanoparticles to be synthesized, thus emerging an antimicrobial agent that can be prepared with optimized properties.

## 4. AgNPs as an Alternative to Combat Human Pathogenic Bacteria

AgNPs have established themselves as a promising alternative to combat different microorganisms. In addition to their ability to inhibit the growth of multidrug-resistant strains, AgNPs exhibit unique characteristics that make them suitable for being useful against these bacteria [18,53,54,55,56,57,58,59,60]. Firstly, among metallic nanoparticles, silver is known for being the most effective nanoparticle against bacteria and other microorganisms, as well as being highly biocompatible and easy to make it function their use in medical applications [37]. A second characteristic is that there are multiple mechanisms associated with its antibacterial activity, where it is suggested that they act on the cell membrane, affecting intracellular components, and altering the respiratory chain [35]. This last one is seen as a major advantage because for bacteria to develop resistance against AgNPs they would have to target multiple mechanisms of action that occur in parallel. It is for these reasons that AgNPs have also been promoted as an alternative to antibiotics [61,62].

In this regard, several studies have been published in recent years covering the possible effects of the use of nanoparticles as an antibiotic agent, where one of the major issues of concern is that AgNPs may induce the appearance of resistant strains. For example, there are articles that demonstrated that after serial and continuous exposure to AgNPs, some bacterial strains exhibit decreased susceptibility to this agent. This is shown in a study carried out with *E. coli* and *S. aureus*, in which the bacteria were exposed to sublethal doses of AgNPs (previously determined in the same study) for 5 days. The results revealed an increase in tolerance to AgNPs by the bacteria, where the half-maximal inhibitory concentration (IC_50_) values increased from 11.89 to 17.59 mg/L in the case of *E. coli*, and from 6.98 to 18.09 mg/L in the case of *S. aureus* [63]. The data suggest that bacteria developed resistance toward a sublethal dose of AgNPs after consecutive selections of the surviving cells.

A similar effect was observed in the research conducted by Panacek et al. [64], in which *E. coli* and *P. aeruginosa* exposed to AgNPs were successively cultured and observed whether the bacteria developed resistance to the nanoparticles. The results revealed that both bacteria became tolerant to the nanoparticles, which was mainly reflected in the value of their MIC that increased from 3.38 mg/L to 13.5 mg/L in *E. coli* after the various rounds of culture, and the same effect was observed in *P. aeruginosa*.

Although these reported cases of resistance to AgNPs were not associated with changes in the genetic material, they do reveal the importance of studying doses and possible mechanisms of resistance or avoidance of resistance. Furthermore, there are other similar previous reports of similar cases of tolerance to AgNPs after previous exposure with *E. coli* and *Bacillus* sp. [65,66].

In addition to the development of tolerance reported in strains subsequently exposed to AgNPs, Kaweeteerawat et al. [63] described in their research that prior exposure of microorganisms to AgNPs could decrease the efficiency of antibiotics. In this case, *E. coli* and *S. aureus* strains previously treated with AgNPs exhibited resistance to ampicillin and other antibiotics, with values from 2 to 8 times higher recorded in MIC compared to those bacteria without previous AgNPs treatment. The study demonstrates that physiological changes, such as membrane thickening and lower permeability, occur in those bacteria that were treated with AgNPs, promoting their posterior resistance to antibiotics.

A similar effect in the generation of tolerance to antibiotics was reported by Kędziora et al. [67], where bacteria were incubated with different concentrations of variated silver nanoformulations consecutively. As a result, strains of *E. coli, Klebsiella pneumoniae*, and *S. aureus* assayed changed their susceptibility to silver nanoformulations, increasing the MIC values after the repeated incubation process with this agent. Additionally, after the long-term exposure to silver nanoformulations, this research also registered reduced susceptibility to antibiotics.

These effects have been observed in recent years, together with the rapid advance in the use of nanomaterials against human pathogenic bacteria. Along with the exact mechanism of action of the nanoparticles, these are topics that continue to be studied to understand the mechanism involved in this acquired tolerance. Nevertheless, in parallel to studies reporting the emergence of resistant strains after treatment with AgNPs, research has also been published demonstrating additive or synergistic effects of AgNPs and different compounds such as plant extracts, polymers, or antibiotics that could mean the development of an alternative treatment to combat multidrug resistant strains, without the risk of promoting the emergence of new resistant strains and avoiding the development of bacteria resistance to AgNPs.

This has been suggested in several studies of the antimicrobial activity of AgNPs combined with antibiotics. In a recent investigation using AgNPs in conjunction with chloramphenicol, kanamycin, ampicillin, among others, it was observed that the treatment with AgNPs + chloramphenicol was able to inhibit the growth of *E. coli*, *S. typhimurium*, and *S. aureus* up to 50%. At the same time, treatment with AgNPs + kanamycin managed to inhibit the growth of these same strains but at a percentage close to 95% [68]. The results pointed out that the joint action is more efficient because the AgNPs alter the integrity and membrane potential, increasing its permeability and allowing the passage of antibiotics more easily. It should be noted that for these trials, sublethal doses of AgNPs and half the minimum inhibitory concentration of antibiotics were used.

The capacity to combat bacterial infections in vivo was probed in research using azlocillin in conjugation with AgNPs against *P. aeruginosa*. Along with evidencing the enhanced antibacterial effect of the antibiotic − AgNPs, this study demonstrated that azlocillin − AgNPs conjugate was able to reduce the colonization of *P. aeruginosa* in the spleen of mouse models [69]. Similarly, Ipe et al. [70] carried out a more complex study in which, in addition to evaluating the combined bactericidal capacity of antibiotics and AgNPs, they evaluated their cytotoxicity to establish biocompatible doses with human fibroblasts. Establishing that the non-toxic dose for this cell line was 1 μg/mL, the bactericidal capacity of 1 μg/mL of AgNPs combined with different antibiotics was tested. Surprisingly, the results showed a bactericidal action even for strains resistant to antibiotics, as was the case for *S. aureus* strains (resistant to ampicillin), while *S. mutans* and *Streptococcus gordonii* strains classified as having intermediate antibiotic sensitivity were shown to be at a “susceptible” level of sensitivity in these assays. Thus, the dose of biocompatible AgNP showed synergistic action with antibiotics of different classes such as for bacterial killing enhancement and in both Gram-positive and Gram-negative bacteria.

Another example is the study of antibacterial activity of AgNPs conjugated with vancomycin (Van) or amikacin (Amk), antibiotics used against Gram-positive and Gram-negative strains, respectively [71]. AgNPs were synthesized by chemical reduction using PVP as a stabilizing agent, and post-synthesis, the antibiotic was aggregated to its union with PVP. The bactericidal activity assayed by the zone of inhibition (ZOI) test showed a synergistic effect of antibiotics and nanoparticles. While the ZOI for increased concentrations of amikacin was 9 mm for *E. coli* and 4–5 nm to *S. aureus*, the ZOI of AgNPs + Amk was 20 mm for *E. coli* and 10 mm for *S. aureus*, being these values approximately double of the ZOI of the antibiotic alone. In the case of vancomycin, no zone of inhibition was observed when applied with the antibiotic alone against *E. coli*, and ZOI of 5–7 mm was obtained for *S. aureus*, meanwhile when using AgNPs + Van against *E. coli* a ZOI of 6 to 8 mm was observed, and a ZOI of 11 mm for *S. aureus*. This study also evidenced positive changes in bacteria susceptibility to antibiotics when used together with AgNPs.

A recent relevant study also evidenced the synergistic effect of nanoparticles and antibiotics, but in this case, the AgNPs were directly synthesized using ampicillin (Amp) as a reducing and capping agent. The antibacterial properties of Amp-AgNPs were evaluated against sensitive and drug-resistant Gram-positive and Gram-negative bacteria [72]. In all cases, the Amp-AgNPs were more effective than ampicillin or chemical synthesized AgNPs, according to their MIC values. The prior result was that the treatment of *P. aeruginosa* multi-drug resistant (MDR) and *K. pneumonia* (MDR) with Amp-AgNPs showed MIC values of 20 μg/mL and 28.12 μg/mL while the MIC values using chemical synthesized AgNPs were in the range of >512 and >640 μg/mL for each bacteria, respectively. It should be noted that the MIC value determined with ampicillin alone was above 720 μg/mL for both bacteria, demonstrating that AgNPs synthesized with a linked antibiotic significantly enhances the antibacterial properties of it. Besides showing a synergistic effect of linking ampicillin to AgNPs, this study showed that the repetitive exposition of bacteria to MIC concentrations of Amp-AgNPs did not cause the emergence of resistant bacteria, positioning itself as an effective alternative for the treatment of multi-resistant bacteria, without causing the appearance of resistance to nanoparticles.

In addition to the use of AgNPs combined with antibiotics, the functionalization or conjugation of AgNPs with different molecules has also been proposed as an effective alternative to obtain high bactericidal activity while avoiding the appearance of resistance in bacteria [73]. With the objective of probing this theory, Ashmore et al. [74] studied the antibacterial effectiveness of uncoated AgNPs vs. coated with PVP (Ag + PVP) and with a synthetic polymer (Ag + Polymer) against *E. coli*. The results of the growth inhibition and MIC assays showed that Ag + Polymer were twice as effective as the AgNPs. Even though the nanoparticles synthesized with the polymer had 10% of the concentration of silver that AgNPs uncoated, this demonstrated that formulations with enhanced antibacterial activity but lower silver ion concentrations needed could be prepared by using polymers as capping agents [74].

Another research shows the synergistic antibacterial effects of green functionalized AgNPs. Using chitosan and brown marine algae extract, they managed to synthesize stable AgNPs with an average size of 12 nm that showed enhanced bactericidal activity against human pathogenic bacteria such as *Salmonella enterica* and *Bacillus cereus*, among others. The antibacterial assays demonstrated that AgNPs prepared with a combination of chitosan and algae extract exhibited higher bactericidal activity, with ZOI values greater than 16 mm for all bacteria tested, while ZOI values of AgNPs or extract alone were lower, between 6 and 12 mm [75]. Following the same line of organic extracts, Murei et al. [76] evaluated the enhanced antibacterial effects of *Pyrenacantha grandiflora* extracts when conjugated with AgNPs and antibiotics (vancomycin, ampicillin, and penicillin). The results showed effective antibacterial activity of *P. grandiflora* tubers acetone extracts, and AgNPs in conjugation with selected antibiotics. Most importantly, MIC assays demonstrated that ampicillin conjugated with AgNPs and plant extract exhibit the lowest value of 0.0064 mg/mL against *K. pneumoniae*, while more than 0.8 mg/mL of ampicillin alone was necessary to inhibit the growth of the same bacteria. In this investigation, it was concluded the synergistic effect of penicillin-AgNPs-methanol extract and of vancomycin-AgNPs-water extract.

Considering the above-mentioned background, resumed in Table 1, a number of advantages of the use of nanoparticles against bacteria can still be mentioned. The main ones being related to the properties of nanoparticles, such as their small size that allows them to cross tissue barriers, solubility, and multiple antibacterial mechanisms of action that reduce the possibility of bacteria developing a specific method of resistance against this nanomaterial.

**Figure 2:**
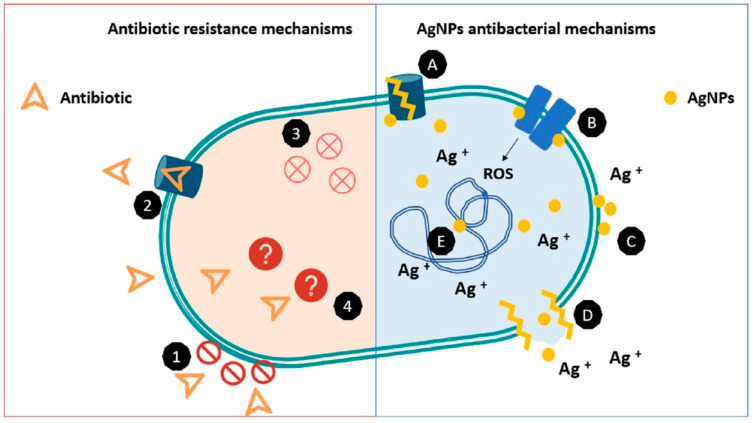
Comparative scheme between resistance mechanisms in bacteria vs antibacterial mechanisms of AgNPs. Antibiotic resistance mechanisms include: (1) permeation barriers, (2) efflux pumps, (3) inactivation of antibiotic, and (4) structural changes in antibiotic targets (represented as “?”) avoiding its recognition. On the other side AgNPs antibacterial mechanisms includes (**A**) alteration of efflux pumps, (**B**) disruption of membrane proteins and electron transport chains (**C**) accumulation in membrane affecting permeation, (**D**) disruption of membrane and leakage of intracellular content, (**E**) interaction and damage in DNA. A similar figure was published in [18].

The strategies that bacteria have acquired that leave antibiotic compounds without effect are well known; these include the acquisition of resistance genes from different bacteria, the formation of biofilms, obstacles to antibiotic permeation, the alteration of the antibiotic target, efflux pump systems that remove the antibiotic in the intracellular media, and others [46]. However, all of these mechanisms do not affect AgNPs action, as was established in Section 3, AgNPs act on bacterial cells by different simultaneous mechanisms that include diffusion of small AgNPs and Ag ions into the cell, disruption and mechanical damage on cell membranes, and alteration of proteins and DNA in the intracellular media [34]. A scheme of these mechanisms is exposed in Figure 2, showing that nanoparticles can exercise their action even if the bacteria have resistance mechanisms. Added to the fact that AgNPs have proven ability to inhibit the formation of biofilms, their antibacterial mechanisms make them suitable for using them in combination with antibiotic or other antibacterial molecules, as they can make the bacteria more vulnerable to these agents by disrupting its membrane, inhibiting biofilm formation, and affecting membrane components such as efflux pumps [18,77].

The fact that the use of AgNPs conjugated with antibiotics in bacterial treatment has not been associated with the appearance of resistance in the existing literature is an important precedent and may be related to the mechanisms of action of these agents [72,73]. While antibiotics act on a specific target in bacterial cells, AgNPs have a more general mechanism of action where they attack multiple cellular structures, and thus, in the combined use of these agents, AgNPs provide the primary interaction and affinity to act and accumulate in cell walls and membranes. This allows concentration of antibiotics, debilitation of bacteria, and even avoidance of already existing mechanisms of resistance since AgNPs can alter and break cellular barriers and membrane proteins, conceding antibiotic the ability to exert its action at a membrane or intracellular level.

The literature supports that the antibacterial action of AgNPs against microorganisms pathogenic to humans is comparable or superior to the activity exerted by antibiotics commonly used to combat such microorganisms (Table 1), which positions AgNPs as an excellent alternative for use in conjunction with or as a replacement for antibiotics. For this reason, along with studies associated with the antibacterial capacity of AgNPs, research has been conducted to evaluate the possible adverse effects that could be generated as a consequence of exposure to AgNPs to establish doses and nanoparticles properties that would be safe for its use in humans.

## 5. In Vitro Toxicity Assays upon Exposure to AgNPs

Since the development of new products and technologies with AgNPs has advanced rapidly, several studies have emerged with the objective of understanding the potential cytotoxic effects of exposure to high doses of AgNPs. In order to establish antecedents about these effects in this section, the in vitro effects of different doses and sizes of AgNPs in mammalian cells will be addressed, considering that the use of medical products containing AgNPs may lead to the ingesta, inhalation, or dermal exposure to this nanomaterial.

### 5.1. Dermic Cell Lines Exposure to AgNPs

The potential consequences of AgNPs in the skin and their penetration have been addressed using cell lines of keratinocytes and dermal fibroblasts principally. Using CRL-2310 cells (human keratinocytes), Sapkota et al. [78] evaluated the effects of exposing this line of keratinocytes to 20 nm AgNPs synthesized from a natural extract. The results of the MTT assay (cell proliferation assay with 3-(4,5-dimethylthiazol-2-yl)-2,5-diphenyltetrazolium bromide) indicate that the effect of nanoparticles on cell proliferation is dose-dependent, obtaining that after 48 h of exposure to 10 μg/mL AgNPs, cell viability was reduced to 98.76%, while after 48 h of exposure to 100 μg/mL viability was reduced to 74.5%.

The same dose-dependent effect was observed in an investigation with HaCaT cells (human keratinocytes) where using AgNPs with diameters of 10, 30, and 60 nm, with different coatings; citrate (CIT), polyethylene glycol (PEG), and bovine serum albumin (BSA) the researchers evaluated cell viability and alterations in cell metabolism after the exposure to different concentrations of AgNPs [57]. It was observed that all nanoparticles at a concentration of 10 μg/mL induced a reduction in the percentage of cell viability to 75–85%. Contrary to what could have been expected, the greatest effects were not observed after the treatment with the smaller nanoparticles but were observed with 40 μg/mL doses of 30 nm nanoparticles coated with citrate after 24 h (50% reduction in cell viability), and with 30 nm nanoparticles coated with BSA after 48 h (30% reduction in cell viability). This established a precedent about how size and coating can influence the effects induced in different cell types, showing that despite its size, the coating in this research was most determinant in the cytotoxic effects. Along with this, the investigation determined that cytotoxicity is associated with changes in HaCaT cell metabolism induced by silver ion release, an action that has been proved is related to the stability and coating of the AgNPs.

The consequences of exposure to nanoparticles have also been studied in human dermal fibroblasts (NHDF) [79]. In this investigation, the results support that the effect on cell viability is related to the size of the nanoparticles used, obtaining a half-maximal effective concentration (EC_50_) of approximately 5 μg/mL with 4.7 nm nanoparticles and an EC_50_ of approximately 2000 μg/mL for 42 nm nanoparticles. On the other hand, it was detected that the nanoparticles induced a significant increase in ROS in a dose-dependent manner.

The cytotoxicity generated by AgNPs coated with tannic acid in 291.03C cells (keratinocytes) was studied by Orlowski et al. [80], evaluating the induction of apoptosis and loss of mitochondrial potential after treatments with concentrations of 1 to 10 μg/mL of 13, 33, and 46 nm AgNPs. The results showed a significant loss of mitochondrial potential of 291.03C cells after exposure to 2.5 μg/mL of 33 and 46 nm nanoparticles. On the other hand, oxidative stress assays did not reveal significant increases in ROS production for any nanoparticle. However, an increase in tumor necrosis factor-alpha (TNF α) production induced by exposure to nanoparticles was observed. Overall, the observed effects were greater for nanoparticles that were not coated with tannic acid. To further investigate these effects, later this research group demonstrated that the increase in TNF α was directly related to the induction of an inflammatory response by AgNPs, that in vivo can promote wound healing and prevent infections [81].

Kaur and Tikoo [82] synthesized AgNPs using tannic acid (TSNPs) and sodium borohydride (BSNPs) and obtained AgNPs with different zeta potential while TSNPs had a size of 30 nm and potential of −34 mV, BSNPs were 50 nm and had a potential of −22 mV. Treatment of skin epithelial cell line A431 with 100 g/mL of TSNPs caused disruption of cell membranes and decrease in cell number, in addition to oxidative stress, however, and as observed in the above-mentioned studies, this effect was dose-dependent. The observation of the cells by TEM revealed that TSNPs were able to enter the cell and accumulate in the cytoplasm and nuclei, while BSNPs were not efficient in entering the cell presumably due to their aggregation because of its low zeta potential. This reveals the influence of size and the stability of AgNPs in the media (in addition to dosage) in the final cytotoxic effects. Adding to the supporting literature, higher cytotoxic effects for smaller nanoparticles.

### 5.2. Respiratory Cell Lines Exposure to AgNPs

The potential effects of inhalation of AgNPs have been addressed in studies using cell lines from the alveolar and bronchoalveolar epithelium. Gene expression profiling analysis of A549 cells (human lung epithelial cell line) after their exposure to 12.1 μg/mL of 15 nm of AgNPs revealed that more than 1000 genes changed their expression after 24 h of exposure to the nanoparticles. These included genes related to cellular stress and genes coding for cell cycle regulatory proteins. The study revealed that the exposure to 12.1 μg/mL of AgNPs generated increased intracellular ROS production and alterations in the cell cycle. However, after 48 h, an increase in the expression of genes related to cell cycle maintenance and regulation was observed, suggesting that during this time, the cells were able to adapt to the exposure to AgNPs [83]. Similarly, Han et al. [84] performed a toxicity study on A549 cells with 15 nm AgNPs synthesized by biological and chemical methods. The MTT assay revealed that 25 μg/mL of the biologically synthesized nanoparticles reduced cell viability by 50% (half maximal inhibitory concentration = IC_50_), whereas for the chemically synthesized nanoparticles, an IC_50_ of 70 μg/mL was obtained. The reduction in cell viability was directly related to the lactate dehydrogenase (LDH) values in the medium, which increased significantly in cultures subjected to AgNP concentrations greater than 20 μg/mL. In this study, both cell proliferation and oxidative stress assays showed a concentration-dependent effect, whereas the differences in inhibitory concentrations between biological and chemical AgNPs can be attributed to the lower stability and higher aggregation of nanoparticles synthesized by chemical method, which reduces their potential cytotoxicity.

Other studies have been performed using human bronchial epithelial cells (BEAS-2B). Gliga et al. [85] investigated the toxicity induced by AgNPs of different sizes (10, 40, and 75 nm) and with different coatings (citrate and PVP). BEAS-2B cells were exposed to doses from 5 to 50 μg/mL of nanoparticles for 4 and 24 h. Cytotoxicity assay determined that only 10 nm nanoparticles (both CIT-AgNPs and PVP-AgNPs) induced toxicity in cells after 24 h of exposure to the highest doses; 20 and 50 μg/mL. Through complementary assays, this investigation also supports the fact that increased cytotoxicity is caused by the smaller AgNPs and that this is likely because they penetrate and release higher concentrations of silver ions in the intracellular medium.

### 5.3. Digestive System Cell Lines Exposure to AgNPs

The toxicity of Ag NPs to liver cells has been studied using C3A cells (human hepatocellular carcinoma-derived cells). One investigation evaluated the effects induced after exposure to 20 nm AgNPs at a concentration of 1.95 μg/ 106 cells. The assays revealed significant deleterious effects on hepatocytes altering their viability and function, with an LC_50_ of 2.5 μg/cm^2^. Toxicity was reflected in increased LDH in the medium and increased inflammatory mediators such as interleukin 8 (IL-8) [86].

Xue et al. [87] also studied the effect of exposure to AgNPs (21.8 nm diameter) on the proliferation and induction of apoptosis of liver cells of the HepG2 cell line (hepatocytes). After 24 h, HepG2 cells exposed to a concentration of 210 μg/mL AgNPs showed only a 10% decrease in cell viability, while assays evaluating apoptosis and ROS generation were performed with different concentrations of nanoparticles (40, 80, and 160 μg/mL). In all cases, a significant increase in both apoptosis and ROS generation was observed, directly related to the concentration of AgNPs to which the cells were exposed. The cytotoxicity induced by the nanoparticles is concluded to be related to an increase in the rate of apoptosis and oxidative stress occurring in a time- and concentration-dependent manner.

The influence of AgNPs coating was evaluated in a study where Hep G2 cells were treated using citrate and PEG-coated nanoparticles. The results of cytotoxic effects showed that coating deeply influences these. While CIT-AgNPs reduced cell viability after being treated for 24 h with 10 μg/mL, PEG-AgNPs reduced cell viability after exposure for 24 h to 5 μg/mL doses. The treatment of HepG2 cells with AgNPs (CIT or PEG) did not induce apoptosis but did downregulate genes related to apoptosis, supporting the hypothesis that the reduction of cell proliferation may be a consequence of necrotic cytostatic or necrotic events [88].

Caco-2 cells (human colorectal adenocarcinoma-derived cell line) have been used to evaluate the potential effects on the intestinal epithelium following nanoparticle ingestion. Abbott Chalew and Schwab studied the effects on cell proliferation and cellular stress resulting from exposure to different concentrations of AgNPs in Caco-2 cells. In their analysis, no significant increase in intracellular ROS was detected after nanoparticle exposure. However, there was an increase in IL-8 production, which reflects the induction of damage and activation of inflammatory responses in Caco-2 cells due to nanoparticle exposure. LC_50_ was determined to be above 100 mg/L, and the effects induced in the cells were dose-dependent [89].

The effect of particle and dose in cytotoxicity was assessed by using the same cell line but different AgNPs. In this research, AgNPs were coated with a small peptide (L-cysteine L-lysine L- lysine) and synthesized at 20 and 40 nm. The exposure of Caco-2 cells to both AgNPs (at 5 to 100 μg/mL) significantly decreased the cell viability after 24 and 48 h, it also induced generation of ROS, but no membrane leakage was detected. The effects were size, dose, and time-dependent. For instance, the treatment with doses of 5 and 10 μg/mL of AgNPs did not reduce the percentage of cell viability beyond 80%, while doses higher than 10 μg/mL decreased to 60% or lower the percentage of cell viability [90].

All the investigations mentioned point common principal effects observed after exposure to AgNPs independent of the cell line studied (Table 2). These are ROS increase and reduction in cell viability, along with the generation of an inflammatory response in some cell lines. Although, in some cases, the exposition causes changes at gene expression levels and metabolism, in general, all the responses are dose-dependent and influenced by the nanoparticle size. Evidencing the parameters that can be controlled or modified in order to reduce the cytotoxic effects of AgNPs.

### 5.4. Epithelial Models to Study In Vitro Effects of AgNPs

The in vitro studies have been established as useful to understand the mechanistic aspects and general factors affecting AgNPs potential cytotoxic effects. However, these investigations do not consider that, in order to get into the organism, the nanoparticles must cross multiple layers of cells and will be exposed to different fluids and pHs. These factors are considered in the in vivo studies. However, these studies involve a series of difficulties associated with working with animal models, as well as with the post-exposure study of tissues and cells. That is why for the study of potential effects and effects induced at cellular levels, a few authors have designed and proposed the use of cultures that mimic the different epithelia in the body.

Using a 3D model named EpiKutis designed to mimic the human epidermis, the evaluation of the cytotoxic and permeation ability of AgNPs in vitro was carried out. The results showed that relative cell viability after the exposure of Epikutis to AgNPs was greater than 80% for all the doses tested (62 to 1000 μg/mL). Meanwhile, penetration assessment revealed penetration percentages of 0.9% when the model was treated with a dose of 100 μg of AgNPs. In this case, AgNPs did not induce oxidative stress, but when using a 2D keratinocytes culture oxidative, stress was observed. Thus, it can be concluded that cells in the 3D model maintained a balance between inflammatory and anti-inflammatory responses induced by AgNPs exposition, presumably this self-regulation resulted in protection against oxidative stress [91]. This is in concordance with the developed applications taking advantage of the healing-promote activity of AgNPs [92,93]. Hence studies using the EpiKutis model might be a more realistic approach to the responses generated as a result of AgNPs exposure in skin.

Similarly, in a more realistic approach, Fizesan et al. [94] created a 3D tetra culture with the aim of analyzing the toxicological effects of AgNPs in the alveolar barrier. Using 20 and 200 nm AgNPs the model was exposed to 0.05, 0.5, and 5 μg/cm^2^ of AgNPs. As expected, AgNPs of 20 nm decreased the cellular viability by more than 20% at the highest dose, but only in the apical compartment of the model. While in the basolateral compartment, a significant decrease of metabolic activity was registered, but not a significant decline in cell viability. An increase of ROS in both compartments was observed after treatments with AgNPs of 20 and 200 nm. Besides showing a dose and size-dependent effect, this research demonstrated that after 24 h, the model was able to recover, returning to basal levels, including the altered gene expression. Proving as Chen et al. [91] research that a 3D model exhibits the self-regulation responses that will be triggered in cells in the organism in order to overcome the possible cytotoxic effects generated by AgNPs exposure.

Although not as a 3D model, a similar approach with the objective of simulating the human gut epithelium, T84 cells (human colon carcinoma derived cell line) were used as an in vitro model for study the effects of AgNPs exposure [95]. This cell line was selected because of its capacity to secrete mucus and emulate the permeation conditions of the epithelium. The exposure treatment was assessed with doses of 20 and 100 μg/mL of AgNPs of 10, 20, 75, and 110 nm. A significant decrease in cell viability was observed only at 100 μg/mL. At the same time, changes in permeability and gene expression were only registered after exposure to 10 nm AgNPs [95]. Supporting previous research has shown that smaller nanoparticles will be able to induce greater damage.

These three studies show that physiological conditions can be emulated in vitro and reflect well the responses that have been observed in in vivo models [96,97,98,99], where lower or null effects in cell viability have been shown, and in direct relation to dose and size of AgNPs (smaller sizes higher cytotoxic effects). At the same time, these models allow easier analysis of the biological and molecular effects.

## 6. Applications of Antibacterial AgNPs in Healthcare

Health and medicine are the sectors with the most research and development of technologies that take advantage of the antimicrobial activity of nanoparticles [19,100]. As a consequence of the biocompatibility and easy functionalization, AgNPs can be applied to different products and give them bactericidal capacity. Here, we review investigations associated with these applications and products of topical use that are currently commercially available.

One main application of AgNPs is their use in face masks to increase their protective ability. Investigations related to this are the face mask coated with nanoparticles synthesized from silver nitrate and titanium dioxide developed by Y. Li et al. [101] that exhibited the ability to reduce up to 100% of *E. coli* and *S. aureus* CFU within 24. In a similar investigation, a commercially available mask was treated with AgNPs solutions at concentrations of 50 and 100 ppm, resulting in masks with incorporated nanoparticles that showed the capacity to inhibit the growth of *E. coli* and *S. aureus* [102]. These results are highly favorable since face masks improved with AgNPs could prevent infections in places such as hospitals where there is a high persistence of pathogenic microorganisms. In addition, a recent study probed the potential of a disinfectant with AgNPs incorporated as an active ingredient to decontaminate surgical masks [103]. The formulation generated shows the high antibacterial activity as well as the mask impregnated with the formulation, being able to inhibit the growth of *E. coli, K. pneumoniae*, and *S. aureus*.

Catheters coated with AgNPs were also a matter of study in order to prevent infections and increase sterility. A study showed that catheters with nanoparticles incorporated were able to inhibit the growth and biofilm formation for at least 72 h of organisms such as *E. coli*, *S. aureus*, *C. albicans*, and managed to significantly reduce the growth of *Enterococcus* and *P. aeruginosa* strains. This research also addressed the possible toxic effects of the catheters in studies with animal models, where the use of coated catheters for up to 10 days did not induce toxic effects or inflammation in the used area. The trial established that up to 84% of the nanoparticle coating remained coated during the period studied, supporting the use of this technology to improve the antimicrobial activity and safety of catheters with nanoparticles incorporated [104].

Similarly, Wu et al. [105] prepared catheters decorated uniformly with AgNPs synthesized by chemical reduction with polydopamine. The product showed effective antibacterial capacity against *S. aureus*, and in specific doses, a good biocompatibility as demonstrated in viability assays with MC3T3 E1 cells (osteoblastic cell line from mouse). In more recent research using an ecofriendly approach to synthesize AgNPs, researchers developed a catheter coated with these nanoparticles that exhibit antibacterial activity against pathogenic microorganisms responsible for urinary tract infections such as *Bacillus* sp., *E. coli*, *K. pneumonia*, *P. aeruginosa*, *S. aureus*, and *Candida albicans* [106]. These studies pave the way to the development of improved catheters with the ability to prevent infections associated with the long time use of this device in hospitalized patients. However, more research related to the biocompatible doses and release rate of AgNPs from the catheters is needed to prevent adverse effects. Nevertheless, there are products already commercially available such as ON-Q Silver Soaker™, Silverline^R^, and AgTive with available varieties of impregnated catheters that are used in different countries.

Besides the antibacterial activity, there is research focused on the healing properties of silver and its nanoparticles that have led to the development of various wound dressings with enhanced healing and antibacterial effects. Tian et al. [107] used a solution of AgNPs for the treatment of superficial wounds, finding that this treatment helped to heal the wounds in a shorter time and with better skin regeneration. It is postulated that this effect is a consequence of the modulating action of AgNPs on pro-inflammatory cytokines. Moreover, it is believed that by decreasing the period of inflammation, AgNPs allow the regeneration process to continue its course faster [108]. Regarding this same property, an investigation created a formula for topical use containing AgNPs with the purpose of being used in dermal wounds such as burns. The result was a formulation with antimicrobial activity without toxic effects and also with the ability to accelerate the wound healing process [109].

Research has also been conducted about AgNPs combined with organic molecules exhibiting better antimicrobial activity. For instance, by using lignin and polyvinyl alcohol, a hydrogel was formulated with AgNPs synthesized in situ. The generated product showed high antimicrobial activity against *E. coli* and *S. aureus*, with almost 100 % of the bacteria killed after 10 h of treatment. This formulation also exhibited good biocompatibility in assays using L929 cells (murine fibroblast cell line) [110]. In another research using methanolic seed extract of *Pongamia pinnata*, AgNPs were prepared to incorporate in a hydrophilic gel. The assays in animal models showed that after 18 days, the wound was reduced after treatment with gel versus 30 days in the not-treated group. Meanwhile, the in vitro assays against pathogenic bacteria showed the gel capacity to inhibit the growth of *E. coli*, *S. aureus*, *B. subtilis*, and *P. aeruginosa* [111]. Like these investigations have been made with AgNPs with chitosan or starch incorporated during their synthesis process, all the formulations generated based on these nanoparticles exhibited healing and antibacterial capacity [81,92,93,112].

Although there are currently cosmetics or drugs with silver as the main component aimed at improving healing, the products generated in the mentioned studies based on AgNPs often show better ability to promote healing and accelerate skin regeneration in wounded areas, demonstrating the superior properties of AgNPs in dressing products destined to the treatment of wounds or prevention of infections. In the same way, the incorporation of AgNPs in hydrogels, catheters, or medical textiles is supported by these investigations in which it is proved that the nanoparticles confer antibacterial properties to the final product.

## 7. Conclusions

**Figure 3:**
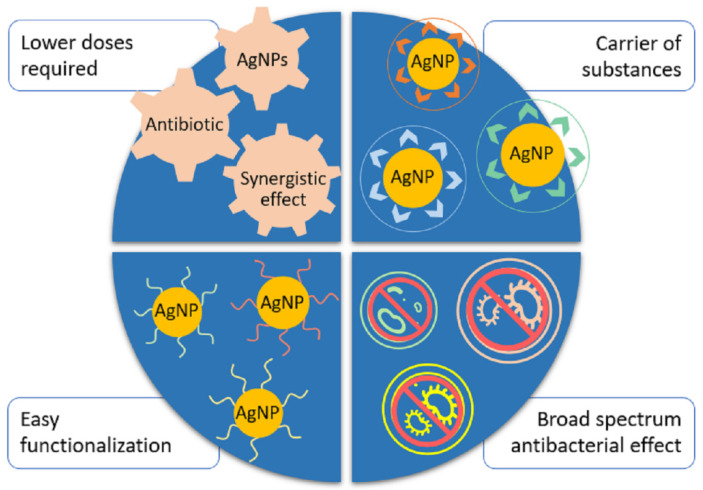
Advantages of using AgNPs in combination or as an alternative to antibiotics.

Nowadays, AgNPs represent an excellent antimicrobial agent and have an exceptional antibacterial capacity. They address many of the criteria to which is considered that new antimicrobial technologies must conform in order to be effective such as antimicrobial performance, fast-acting, and low cytotoxicity; and finally, nanoparticles can be manipulated in order to achieve selectivity and delivery to specific targets [62]. Their use against bacteria must be regulated and measured, avoiding unnecessary exposure of microorganisms to sublethal doses of nanoparticles that could promote the development of tolerance to this agent. The main advantages of the use of nanoparticles in conjunction with antibiotics are shown in Figure 3. The use of AgNPs reduces the necessary doses of the antibiotic and the nanoparticle required to achieve an effective antibacterial activity against various bacteria, thus decreasing the likelihood of side effects [18]. Nanoparticles can form complexes to act as carriers of drugs or antibiotics [113], improving their release and selectivity, nanoparticles can be functionalized with different molecules in order to improve their antibacterial effect [75,76], and finally, they exert antibacterial activity on various different bacterial types that include Gram-negative and Gram-positive bacteria, and resistant strains (Table 1) [33,53].

The knowledge of the effects at a cellular level and the study of the effects in the long term are essential issues to investigate in order to determine the dosage and safety of AgNPs. Knowing the behavior of AgNPs in biological media and in combination with other materials will provide the necessary information to develop new technologies and nanomaterials with the ability to prevent infections or eliminate selectively pathogenic bacteria. Since it is known that properties such as size and coating will affect AgNPs toxicity, their functionalization may reduce or prevent cytotoxic effects associated with AgNPs exposure and, at the same time, increase their antibacterial capacity and selectivity. These data support the use of AgNPs as antibacterial; however, appropriate strategies for their use must be studied and developed without aggravating the situation of the emergence of resistant strains.

## Figures and Tables

**Table 1:** Resume of research addressing the use of AgNPs combined with antibiotics.

Antibiotic Used with AgNP	Bacteria Tested	Antibacterial Parameters	Reference
Chloramphenicol, kanamycin, biapenem, aztreonam, ampicillin.	*E. coli*, *S. typhymurium* *S. aureus*, *B. subtilis*	Additive and synergistic effect of combined treatment of AgNPs + Chloramphenicol and AgNPs + kanamycin according to FICI ^1^	[68]
Azlocillin	*P. aeruginosa*	AgNPs conjugated with azlocillin enhanced antibacterial activity from MIC = 8 ppm for azlocillin alone to MIC = 4 ppm for AgNPs + azlocillin,	[69]
Erythromycin, ampicillin, chloramphenicol, cephalothin, clindamycin, tetracycline, gentamycin, amoxicillin, ciprofloxacin, cefpodoxime, cefuroxime	Multi resistant *S. aureus* (MRSA), *S. mutans*, *S. oralis*, *S. gordonii*, *Enterococcus faecalis*, *E. coli*, *A. actinomycetemcomitans*, *P. aeruginosa*	Antibacterial effectiveness of antibiotics increased synergistically from no growth inhibition into the susceptible range when combined with AgNPs	[70]
Vancomycin, amikacin	*E. coli*, *S. aureus*	AgNPs functionalized with antibiotics showed synergistic antibacterial effects. Going from resistant to vancomycin to susceptible in the case of *E. coli*.	[71]
Ampicillin	*E. coli*, *S. aureus*, *Ampicillin resistant E. coli*, *Ampicillin resistant S. aureus*, *K. pneumonia* (MDR) and *P. aeruginosa* (MDR)	AgNPs synthesized with ampicillin. MIC 3 to 28 μg/mL AgNPs-Amp against all bacteria tested vs. 12 to >720 μg/mL of ampicillin alone.	[72]
Ampicillin	*E. coli*, *E. coli ampicillin resistant*, *P. aeruginosa ampicillin resistant*, *E. aerogenes ampicillin resistant*, *V. cholerae* and *S. aureus* (MRSA)	AgNPs functionalized with ampicillin reduced the CFU in all bacteria tested, even resistant strains.	[73]
Vancomycin, ampicillin, penicillin	*S. aureus, E. coli, K. pneumoniae*	Conjugated Ampicillin with AgNPs effective against all bacteria. All antibiotics increase antibacterial activity when conjugated with AgNP	[74]

^1^ FICI = fractional inhibitory concentration index.

**Table 2:** Resume of cytotoxic effects registered in mammalian cell lines after exposure to different doses of AgNPs.

Coating and Size of AgNP	AgNP Dosage	Cell Type	Cytotoxic Effect	Reference
20 nm	10 to 100 μg/mL	CRL-2310	Dose-dependent effect in cell viability reduction. Viability of 98.76% after treatment with 10 μg/mL	[78]
10, 30 and 60 nm CIT, PEG, BSA	0 to 100 μg/mL	HaCaT	Alterations in metabolism and energy production related to ROS increase.	[57]
4.7 and 42 nm	5 to 2000 μg/mL	NHDF	ROS increase, reduction of cell viability dose and size-dependent.	[79]
13, 33 and 46 nm tannic acid	1 to 10 μg/mL	291.03C	Dose-dependent reduction in cell viability. Up regulation of TNF α	[80]
30 and 50 nm tannic acid and sodium borohydride	5 to 100 μg/mL	A431	Dose-dependent decrease of metabolic activity. Up regulation of TNF α	[82]
15.9 nm	12.1 μg/mL	A549	Modification in gene expression, increase in ROS production	[83]
15 nm	0 to 50 μg/mL	A549	Reduction in cell viability, increase in ROS dose dependent	[84]
10, 40 and 75 nm PVP, CIT	5 to 50 μg/mL	BEAS 2B	Toxicity only at 20 and 50 μg/mL of 10 nm AgNP, damage in DNA.	[85]
20 nm	1.95 μg/10^6^ cells	C3A	Reduction in viability and cell function. Increase in IL-8 and TNF α	[86]
21.8 nm	0 to 1600 μg/mL	HepG2	Increase ROS production in dose dependent manner. Reduction of cell viability.	[87]
30 nm CIT, PEG	0 to 50 μg/mL	HepG2	Changes in expression of genes related to apoptosis and cell cycle.	[88]
200 nm	0 to 100 μg/mL	Caco 2	Significant toxic effects only at 100 μg/mL. Increase in IL-8 production.	[89]
20 and 40 nm peptide coated	5 to 100 μg/mL	Caco 2	Reduction of cell viability, increase in ROS, dose and size-dependent.	[90]
